# Progesterone boosts abiraterone-driven target and NK cell therapies against glioblastoma

**DOI:** 10.1186/s13046-024-03144-2

**Published:** 2024-08-06

**Authors:** Hsien-Chung Chen, Hong-Yi Lin, Yung-Hsiao Chiang, Wen-Bin Yang, Chung-Han Wang, Pei-Yu Yang, Siou-Lian Hu, Tsung-I Hsu

**Affiliations:** 1 Ph.D. Program in Medical Neuroscience, College of Medical Science and Technology, Taipei Medical University and National Health Research Institutes, Taipei, Taiwan; 2 Taipei Neuroscience Institute, Taipei Medical University, Taipei, Taiwan; 3 Department of Neurosurgery, Shuang Ho Hospital, Taipei Medical University, Taipei, Taiwan; 4 Research Center for Neuroscience, Taipei Medical University, Taipei, Taiwan; 5 Department of Neurosurgery, Taipei Medical University Hospital, Taipei Medical University, Taipei, Taiwan; 6 Department of Surgery, College of Medicine, Taipei Medical University, Taipei, Taiwan; 7 International Master Program in Medical Neuroscience, College of Medical Science and Technology, Taipei Medical University, Taipei, Taiwan; 8 TMU Research Center for Drug Discovery, Taipei Medical University, Taipei, Taiwan; 9 Ph.D. Program in Drug Discovery and Development Industry, College of Pharmacy, Taipei Medical University, Taipei, Taiwan; 10 TMU Research Center of Cancer Translational Medicine, Taipei, Taiwan

## Abstract

**Introduction:**

Glioblastoma (GBM) poses a significant challenge in oncology, with median survival times barely extending beyond a year due to resistance to standard therapies like temozolomide (TMZ). This study introduces a novel therapeutic strategy combining progesterone (Prog) and abiraterone (Abi) aimed at enhancing GBM treatment efficacy by modulating the tumor microenvironment and augmenting NK cell-mediated immunity.

**Methods:**

We employed in vitro and in vivo GBM models to assess the effects of Prog and Abi on cell viability, proliferation, apoptosis, and the immune microenvironment. Techniques included cell viability assays, Glo-caspase 3/7 apoptosis assays, RNA-seq and qPCR for gene expression, Seahorse analysis for mitochondrial function, HPLC-MS for metabolomics analysis, and immune analysis by flow cytometry to quantify NK cell infiltration.

**Results:**

Prog significantly reduced the IC50 of Abi in TMZ-resistant GBM cell, suggesting the enhanced cytotoxicity. Treatment induced greater apoptosis than either agent alone, suppressed tumor growth, and prolonged survival in mouse models. Notably, there was an increase in CD3^−^/CD19^−^/CD56^+^/NK1.1^+^ NK cell infiltration in treated tumors, indicating a shift towards an anti-tumor immune microenvironment. The combination therapy also resulted in a reduction of MGMT expression and a suppression of mitochondrial respiration and glycolysis in GBM cells.

**Conclusion:**

The combination of Prog and Abi represents a promising therapeutic approach for GBM, showing potential in suppressing tumor growth, extending survival, and modulating the immune microenvironment. These findings warrant further exploration into the clinical applicability of this strategy to improve outcomes for GBM patients.

**Supplementary Information:**

The online version contains supplementary material available at 10.1186/s13046-024-03144-2.

## Introduction

Glioblastoma (GBM) represents one of the most formidable challenges in the realm of neuro-oncology, characterized by its aggressive growth and profound resistance to existing therapeutic regimens [1, 2]. Despite decades of research and significant advancements in cancer treatment, the prognosis for GBM patients remains bleak, with median survival times barely extending beyond a year post-diagnosis, primarily due to the acquisition of resistance to temozolomide (TMZ) [3–5]. This reality underscores an urgent need for innovative approaches capable of penetrating the complex molecular fortress presented by GBM. Our previous investigations have shed light on the critical role of Cytochrome P450 (CYP) 17A1-dependent steroidogenesis in contributing to the resilience of GBM against chemotherapeutic interventions [6, 7]. In particular, abiraterone (Abi) acetate, a FDA-approved CYP17A1 inhibitor for prostate cancer, and its derivatives exhibit the significantly suppressive efficacy on GBM with or without TMZ resistance [6, 7]. These findings provide the clue for exploring novel therapeutic targets that circumvent traditional resistance mechanisms.

The potential of progesterone (Prog) in this context emerges as a beacon of hope. Distinguished from CYP17A1-dependent steroids, Prog offers a dual advantage: a metabolic pathway independent of CYP17A1 expression and a plethora of neuroprotective effects that extend beyond tumor suppression [8, 9]. Our research has demonstrated that Prog metabolite, allopregnanolone, can significantly impede the proliferation and invasion of GBM cells [10]. The ability of Prog to traverse the blood-brain barrier (BBB) efficiently, positions it as a potent candidate for GBM treatment. The therapeutic effects of Prog on GBM present a complex landscape with contradictory findings highlighting the necessity for further investigation. While one study demonstrates potential of Prog in synergistically enhancing the cytotoxic effects of TMZ on GBM cells and mitigating side effects [11], another reveals Prog to promote GBM cell invasion through mechanisms involving MMP-9 and cSrc kinase activation [12]​​​​. These divergent outcomes underscore the importance of Prog administration in GBM to potentially harness its benefits while mitigating adverse effects on tumor progression.

GBM is well-known to enrich an immune-suppressive microenvironment through recruiting tumor-associated macrophage, myeloid-derived suppressor cells, and T regulatory cells. In addition, GBM cells are able to induce the exhaustion of T and NK cells through increasing TGFβ secretion and PD-L1 expression [13, 14]. Particularly, T and NK cell infiltration is correlated with favoured prognosis even though BBB is disrupted variably in GBM patients [15]. Particularly, NK cells were shown to kill EGFRvIII-and HER2-positive GBM [16]. Therefore, NK cell therapy holds promise for treating GBM if we could overcome GBM-released inhibitory signals against NK cells. Prog has been noted for its role in modulating the tumor microenvironment in breast cancer, including influencing immune cell infiltration and differentiation [17]. Prog-conjugated therapy was shown to enhance CD8^+^ T cell infiltration accompanied by the reduction in tumor size of breast cancer [18]. Moreover, stromal expression of the Prog receptor (PgR) correlates with therapeutic efficacy in endometrial cancer [19]. However, the action of Prog in the brain and other hormone-sensitive organs differs. Consequently, it remains unclear whether Prog is involved in shaping the immune microenvironment in GBM. Importantly, reduced T cell infiltration correlates with the poor response of prostate cancer patients to Abi treatment [20], suggesting that a Prog-shaped immune microenvironment might enhance the tumor-suppressive efficacy of Abi. This uncertainty underscores the need to investigate the potential effects of Prog on immune responses within the GBM context.

Although Abi and Prog individually exhibit suppressive effects on GBM, it remains unclear if their combination offers additional benefits for GBM patients. Furthermore, their impact on the immune microenvironment is unknown. Therefore, we aim to elucidate whether manipulating steroid synthesis through Abi and Prog can initiate a tumor-suppressive immune response, particularly enhancing NK cell activity against GBM cells. This investigation could reveal novel insights into the potential synergistic effects of Abi and Prog, offering new therapeutic strategies for GBM treatment. In addition, the study on the impact of medroxyprogesterone on glial tumor growth presents a compelling case for the value of Prog in GBM management [21, 22]. Therefore, the exploration how Prog enhances NK cell-mediated therapies, in particular, opens a new frontier in the fight against this relentless disease.

## Materials and methods

### Chemicals and cell culture

GBM cells were maintained as described previously [23–26]. U87MG-luc, A172, and T98G cells were purchased from ATCC (Manassas, VA, USA). GL-261 cells were purchased from Creative Bioarray (Shirley, NY, USA). U87MG-luc, A172-luc and MGMT-positive T98G cells were maintained in the DMEM supplemented with 10% fetal bovine serum and 100 µg/ml penicillin/streptomycin. For A172-luc, before NK-related experiments, A172 cells were infected with luciferase-expressed-lentivirus for 72 h. Pt#3 cells were isolated from a GBM patient of Shuang-Ho Hospital. The patient consent and isolation protocol were described in the previous studies [23, 25, 27]. TMZ-resistant GBM cells were established as described previously [25, 28]. TMZ-resistant U87MG-luc-R, Pt#3-R, and GL261-R cells were maintained in the presence of 100 µM of TMZ (MilliporeSigma Corporate, St. Louis, MO, USA) [7, 24]. NK-92 cells were maintained in the EL-873 serum-free medium supplemented with EliteMu™ (EliteCell Biomedical Corp., Woodway, TX, USA) and IL2 (Croyez Bioscience Co., Ltd., Taipei Taiwan). Viability of NK-92 cells was estimated once per three days using FACSCOPE B Cell Counter (Curiosis Inc., Seoul, Republic of Korea). Progesterone and abiraterone acetate were purchased from Cayman Chemical (Ann Arbor, MI, USA).

### Cell viability assay and glo-caspase 3/7 assay

Cell viability was estimated using CCK8 assay as described previously [7, 29]. Cultured medium was collected and 10 µl of medium was mixed with the reagent of Glo-caspase 3/7 assay kit (Promega Corporation, Madison, WI, USA), followed by the estimation of GloMax^®^ Discover Multimode Reader (Promega Corporation). After incubation with NK-92 cells, viability of U87MG-luc, U87MG-luc-R and A172-luc cells was estimated by GloMax^®^ Discover Multimode Reader.

### Synergism evaluation

Synergistic effect on cell viability was analysed using the CompuSyn software. CI < 1 was defined as the synergistic effect [30, 31].

### Intracranial implantation with GBM cells and NK cell therapy

Animal experiments were approved by the institutional animal care and use committee of Taipei Medical University (LAC-2020-0303, LAC2022-0314 and SHLAC2023-0096). NOD.CB17-Prkdcscid/NCrCrl mice (8-week-old) and C57BL/6 mice (8-week-old) were purchased from BioLASCO Taiwan Co., Ltd. (Taipei, Taiwan). The protocol was described in our previous studies [7, 23, 24]. Briefly, GBM cells (5 × 10^5^) in 5 µl medium was collected and implanted into the brain on Day 0 using stereotaxic instrument and micro-injector. Drugs were injected intraperitoneally on Day 6 (once per three days) until the death of mice. For IVIS image, mice were injected with 200 µl luciferin (15 mg/ml; R&D Systems, Inc., Minneapolis, MN, USA) intraperitoneally, and then subjected to image establishment by IVIS Lumina III XRMS (PerkinElmer, Waltham, MS, USA). To evaluate the suppressive effect of NK-92 in GBM initiation, U87MG-luc or U87MG-luc-R cells (5 × 10^5^) were mixed with NK-92 cells (5 × 10^5^) in 5 µl medium, followed by the intracranial implantation.

### Histological analysis and immunostaining

Brain tissues were excised from the experimental mice, and were fixed in the 4% paraformaldehyde, followed by the paraffin-embedded slides (10 μm) preparation. After antigen retrieval, slides were immunostained using VECTASTAIN^®^ ABC Kit (Newark, CA, USA) [23]. In particular, the anti-NKG2D and PCNA antibodies were purchased from Bioss Inc. (Woburn, MS, USA) and Cell Signaling Technology, Inc. (Danvers, MA, USA), respectively.

### Human specimens and RNA-seq

Specimens of GBM patients including primary and recurrent GBM were acquired as described previously [24]. RNA-seq of specimens was performed with the assistance of Biotools Inc [24].

### Bioinformatics analysis

NK cell infiltration in GBM was estimated using the TIMER website (http://timer.cistrome.org/). Patient prognosis and the correlation of gene expression in GBM was estimated using the Gliovis website (http://gliovis.bioinfo.cnio.es/).

### Western blotting

The protocol was described previously [23, 24, 26]. Briefly, blocked PVDF membrane (MilliporeSigma Corporate) was incubated with the anti-MGMT (ABclonal, Woburn, MA, USA), anti-GAPDH (GeneTex International Corporation, HsinChu, Taiwan), anti-cyclin A2 (Abcam, Cambridge, UK), anti-CDK1 (Abcam) or anti-α-tubulin (GeneTex) at 4℃ overnight, followed by the incubation with the HRP-conjugated secondary antibody at room temperature for 1 h. ECL-reacted membrane was subjected to image analyser (MilliporeSigma Corporate).

### RT-qPCR

RNA was extracted using the TRIsure reagent (Meridian Bioscience, Cincinnati, OH, USA) and RNA extraction kit. Reverse transcription was performed using the reverse transcriptase kit (Takara Bio Inc., Shiga Japan). SYBR green for qPCR was purchased from Promega Corporation. For qPCR, mixture of cDNA, primers and SYBR green was analyzed by the PCR machine. Primers: ALDOC (F)-gccctgagtgaccatcatgtat; ALDOC (R)- tgaatgatgcctcttcttcgct. PC (F)-gcctggagtataagcccatcaa; PC (R)- gcactgcatctacgttgttctc. IDH3G (F)-gaacaaacaattcctccgtccg; IDH3G (R)- gtggcaggttatggttggtttc.

### MGMT promoter methylation analysis

T98G and U87MG-R cells were seeded at a density of 5 × 10^4^ cells per 60 mm dish and cultured overnight. Subsequently, forty µM Prog was added to the culture medium. Cells were collected at 0, 48 and 72 h post-treatment. Genomic DNA (gDNA) was extracted using the Wizard^®^ Genomic DNA Purification Kit (Promega, WI, USA) according to the manufacturer’s instructions. One µg of gDNA was utilized for DNA methylation analysis, which was performed by Genomics BioSci & Tech. Co., Ltd (New Taipei City, Taiwan). Briefly, genomic DNA was processed with a bisulfite solution to convert the unmethylated cytosines in DNA to uracil. CpGs methylation are tested with the PyroMark Q24 CpG MGMT kit provided by Qiagen [32, 33]. CpG sites in exon 1 of the human *MGMT* gene (genomic sequence on chromosome 10 from 131,265,507 to 131,265,534: cgctttgc gtcccgacgc ccgcaggtc. The purified templates were incubated with specific sequencing primer and run in the Qiagen Pyromark Q24 system. The result was analyzed with PyroMark Q24 software.

### Metabolic analysis targeting glucose metabolism by UPLC-MS

Tis experiment was performed with the assistance of Biotools Inc. (New Taipei City, Taiwan). Cell pellets were mixed with 1 mL 80% methanol for protein precipitation. Samples were centrifuged with 12,000 g for 10 min at 4 °C. Supernatant was dried with nitrogen gas. Residue were dissolved with 200 µL water with internal standard. Samples were analyzed using Waters ultra-high-performance liquid chromatography coupled with Waters Xevo TQS MS (Waters Corp., Milford, MS, USA). MS was operated in negative and positive with multiple reaction monitoring mode. Major MS/MS fragment patterns of each analyte were determined with tuning method. The optimized parameters were as follows: capillary voltage at 1 kV; desolvation temperature at 500 °C; source temperature at 150 °C; and gas flow at 1000 L/h. The chromatographic separation was achieved on a BEH C18 (100 × 2.1 mm, particle size of 1.7 μm; Waters Corp.) at 45 °C with elute A (water with 10 mM tributylamine and 15 mM acetic acid) and eluent B (50% acetonitrile with 10 mM tributylamine and 15 mM acetic acid), and the flow rate was set at 0.4 mL/min.

### Seahorse XF cell mito stress test

The detailed protocol was described in the previous study [24]. Briefly, the assay was performed according to the instruction from the XFe24 Seahorse Mitochondrial Respiration Mito Stress Test (Agilent Technologies, Inc., Santa Clara, CA, USA).

### Seahorse glycolysis stress test

Cells (2 × 10^4^ per well) were seeded into the Seahorse XF24 Islet Capture Microplate and incubated at 37℃/5% CO2 overnight. Before the estimation by the Seahorse analyser (Agilent Technologies, Inc.), cells were incubated with DMEM supplemented with 2% FBS in the absence of glucose and NaHCO3 for 1 h at 37℃ without CO2.

### Immuno-analysis

The protocol in the previous study was followed [34]. Briefly, brain tissue containing implanted GBM cells was excised and homogenized in the iced HBSS supplemented with 10% FBS (Biological Industries, Kibbutz Beit-Haemek, Israel). Purified cells were immune-reacted with the antibody against immune cell markers, including anti-CD3 (Thermo Fisher Scientific, Waltham, MA, USA), anti-CD19 (Thermo Fisher Scientific), anti-NK1.1 (Thermo Fisher Scientific), anti-MHCII (Thermo Fisher Scientific), anti-CD16/CD32 (Thermo Fisher Scientific), anti-CD4 (Thermo Fisher Scientific), anti-CD11c (BioLegend, San Diego, CA, USA), anti-CD8 (BioLegend), anti-CD14 (BioLegend), anti-CD62L (BioLegend) and anti-CD25 (BioLegend) antibodies. After washing, samples were analysed using flow cytometry (Thermo Fisher Scientific).

### PI staining and flow cytometry for cell death assay

T98G or U87MG cells were seeded at a density of 3 × 10^5^ cells per well in a 6-well plate. Once the GBM cells had fully adhered, NK cells (1.5 × 10^5^) were added per well. Simultaneously, varying concentrations of Prog were administered. The cells were then incubated at 37 °C for 18 h. After incubation, the cells were harvested and stained with 1 µg/mL of propidium iodide (PI, P4170, Sigma-Aldrich, MO, USA) for 30 min, protected from light. The stained cells were subsequently analyzed using flow cytometry on a Guava^®^ easyCyte™ Flow Cytometer, and the data were processed with the Guava^®^ easyCyte System 3.3 (Cytec Industries Incorporated, NJ, USA).

### Statistical analysis

The experiments were conducted independently three times, and the results are presented as the mean ± standard error of the mean (SEM). A *P* value of less than 0.05 indicated a statistically significant difference. Survival rates were compared using the log-rank test. For comparisons between two groups, Student’s *t* test was employed. When comparing two groups at various time points, a two-way analysis of variance (ANOVA) was utilized.

## Results

### Synergy between prog and abiraterone (Abi) in GBM

Our prior investigations revealed CYP17A1-targeted Abi as a potent inhibitor of GBM survival, primarily through the induction of endoplasmic reticulum (ER) stress [6]. Nonetheless, the application of Abi in TMZ-resistant GBM remains hindered by a lack of understanding of its underlying mechanisms. Additionally, our findings highlighted that allopregnenolone, a metabolite of Prog, when used in conjunction with TMZ, synergistically inhibits the proliferation of GBM cells [10]. Given these insights, our current study aimed to explore the enhanced therapeutic efficacy of combining Abi with Prog in the treatment of TMZ-resistant GBM.

**Fig. 1:**
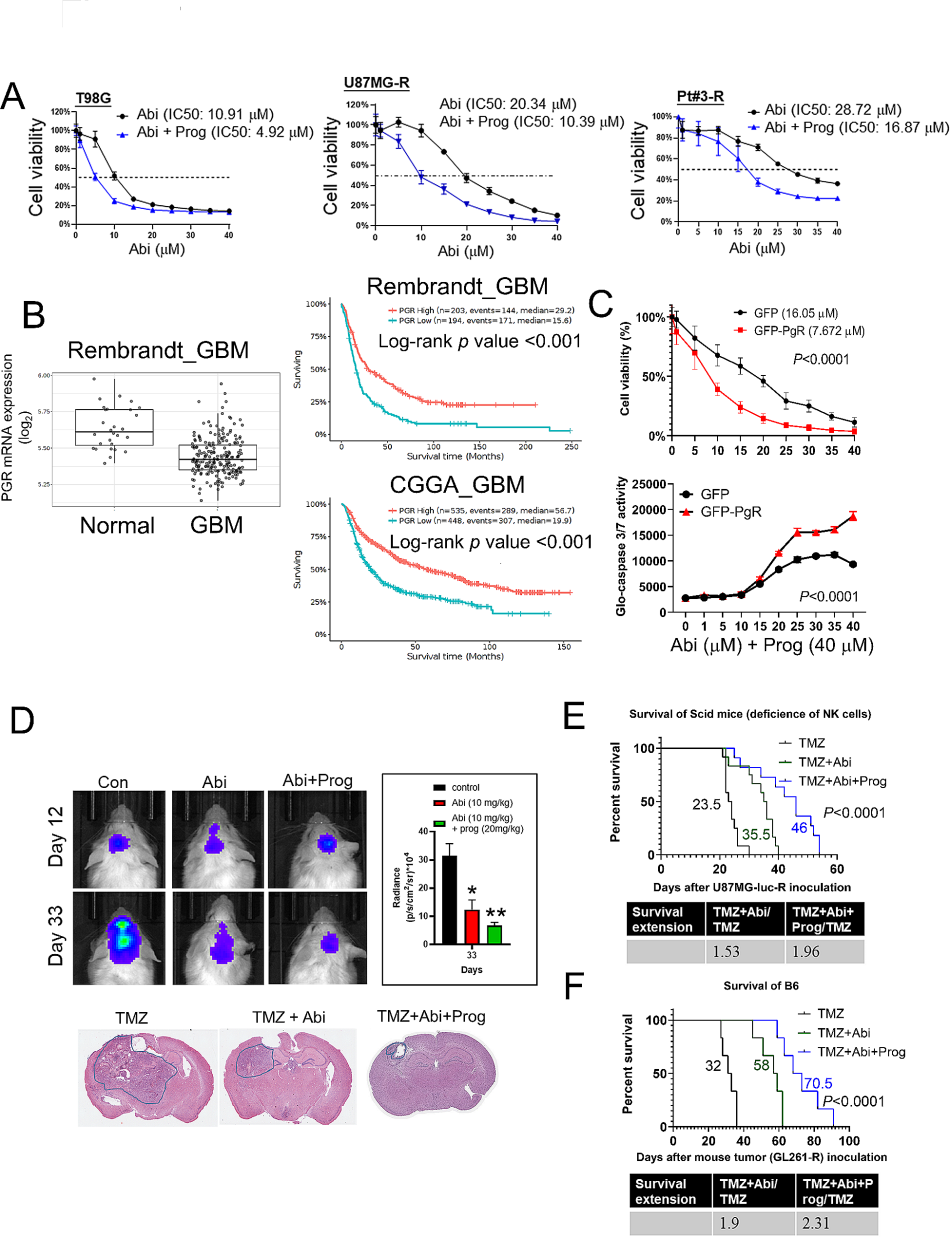
Therapeutic efficacy of Abi combined with Prog on GBM in vitro and in vivo. **A** After treatment with Abi with or without 40 µM Prog for 72 h, cell viability was estimated using CCK8 assay. IC50 was calculated using the Prism software. Experiments, each containing three replicates, were performed independently three times. **B** Clinical relevant of PGR expression was analysed using the Gliovis website. **C** Upper: After transfection with GFP or GFP-PgR for 24 h, Pt#3-R cells were treated with Abi with or without Prog for 72 h, followed by the CCK8 assay. Lower: Medium were collected and analysed using the Glo-caspase 3/7 assay. GFP and GFP-PgR groups were compared statistically using two-way ANOVA. **D** Upper: After intracranial implantation with U87MG-luc-R, tumor growth was detected using IVIS image. Abi and Prog were injected intraperitoneally on Day 6 (once per three days). Lower: Mouse brain was subjected to paraffin-embedded slides preparation, followed by the HE staining. TMZ: 12 mice; TMZ + Abi: 12 mice, TMZ + Abi + Prog: 11 mice. **E-F**. Log-rank test was used to performed survival comparison was performed in Scid **E** receiving U87MG-R-luc implantation and wild type C57BL/6 **F** mice receiving GL-261-R implantation. For the experiment of C57BL/6 mice. **F** Six mice were included for each group. Survival extension days by Abi and Abi + Prog was shown

Initially, we determined the half-maximal inhibitory concentration (IC50) of Abi on various TMZ-resistant GBM cell lines, both with and without the addition of Prog. The incorporation of Prog significantly reduced the IC50 values of Abi across all tested cell lines: in O^6^-methyl-guanine-DNA methyltransferase (MGMT)-positive T98G cells from 10.91 to 4.92 µM, in U87MG-R cells from 20.34 to 10.39 µM, and in Pt#3-R cells from 28.72 to 16.87 µM **(**Fig. 1A**)**. Interestingly, Prog alone did not influence cell proliferation, indicating its role in boosting the efficacy of Abi rather than exerting a direct antiproliferative effect on its own. (Supplementary Figure **S1**). These outcomes underscore the capacity of Prog to amplify Abi-mediated inhibitory effect on GBM growth. Notably, Prog receptor (PgR) expression was markedly lower in GBM specimens compared to normal brain tissues, and high PgR expression significantly correlated with better prognosis **(**Fig. 1B**)**, indicating that PgR expression may benefit the prognosis of GBM patients. Therefore, further investigation focused on the role of the PgR in mediating the action of Prog. Overexpression of GFP-tagged PgR in Pt#3-R cells not only bolstered the impact of Prog on reducing cell viability but also led to a marked induction of apoptosis, as evidenced by increased caspase 3/7 activity **(**Fig. 1C**)**. To assess the synergy between Abi and Prog in treating GBM, we analyzed the combination index (CI) using CompuSyn software [30, 31, 35]. The results showed CI values of less than 1 for the synergistic treatments in various GBM cell lines: Abi at 5–35 µM with Prog (40 µM) in T98G cells, 1–40 µM with Prog (40 µM) in Pt#3-R cells, and 5–35 µM with Prog (40 µM) in U87MG-R cells (Supplementary Figure **S2**). These findings indicate a synergistic interaction between Abi and Prog in GBM treatment. Notably, in an orthotopic brain tumor mouse model, co-administration of Abi and Prog significantly curtailed the progression of implanted U87MG-R-luc GBM tumors **(**Fig. 1D**)**. Survival analyses revealed that Abi monotherapy extended the lifespan of mice implanted with U87MG-luc-R and GL-261-R GBM cells, in both Scid and C57BL/6 wild-type mice **(**Fig. 1E and F**)**. Remarkably, the addition of Prog to the treatment regimen further prolonged survival times significantly: from 23.5 to 46 days in Scid mice, and from 32 to 70.5 days in C57BL/6 mice, achieving survival extension ratios of 1.96 and 2.31, respectively **(**Fig. 1E and F**)**. These findings not only demonstrate the superior therapeutic efficacy of the Abi and Prog combination in GBM treatment, particularly in C57BL/6 mice with a functional immune system but also suggest that modulation of the immune microenvironment may play a crucial role in the effectiveness of this therapeutic approach.

### Prog reduces MGMT expression through decreasing protein stability

**Fig. 2:**
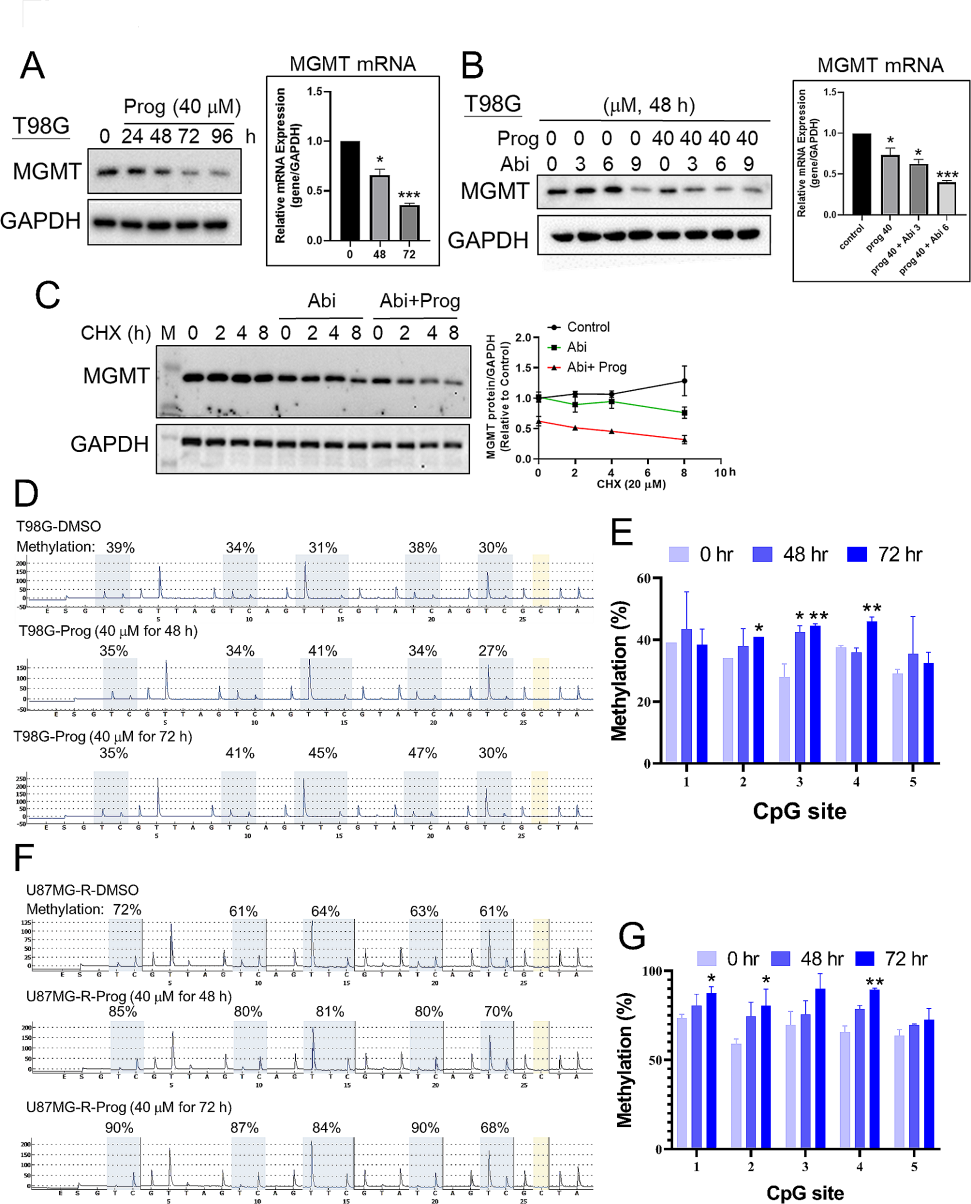
Effect of Prog on MGMT expression. **A**, **B** After treatment with Prog in the absence and presence of Abi, T98G cells were harvested for Western blotting (Left) and RT-qPCR (Right). **C** Left: After treatment for 48 h, T98G cells were treated with 20 µM cycloheximide (CHX) for the indicated time interval, and cell lysates were collected for Western blotting. Right: Quantitative results. **D** After treatment with Prog, gDNA of T98G and U87MG-R cells **F** was purified, and subjected to pyrosequencing analysis. The percentage of methylation in 5 CpG residues in T98G and U87MG-R cells were quantified. Experiments were performed independently three times. (*P < 0.05, **P < 0.01)

In Fig. 1A, the data revealed that MGMT-positive T98G cells displayed a notably lower tolerance to the co-treatment of Abi and Prog, prompting an investigation into MGMT expression. MGMT plays a pivotal role in the development of TMZ resistance in GBM [36], making this line of inquiry particularly relevant. We demonstrated that treatment with Prog for 72 h resulted in a significant dose-dependent reduction in both protein and mRNA levels of MGMT in T98G cells **(**Fig. 2A**)**. Interestingly, while Abi alone did not affect MGMT expression, its combination with Prog not only amplified the reduction in MGMT mRNA and protein levels but also notably decreased the protein stability of MGMT after 48 h of treatment **(**Fig. 2B and C**)**. It is well-known that the methylation status of the MGMT promoter plays a crucial role in controlling its expression level in GBM. To investigate whether Prog influences MGMT promoter methylation, we treated T98G cells with Prog for 72 h and observed an increase in methylation at three CpG residues using pyrosequencing [32, 33] **(**Fig. 2D-E**)**. In MGMT-negative U87MG-R cells [26], the MGMT promoter remained highly methylated, and Prog further enhanced this methylation **(**Fig. 2F-G**)**. These results suggest that Prog may decrease MGMT expression by increasing the promoter methylation of MGMT. This synergistic effect between Abi and Prog also suggests a potent therapeutic strategy for targeting MGMT-positive tumors, underscoring the potential of this combined treatment in overcoming TMZ resistance in GBM.

### Abi decreases mitochondria activation in TMZ-resistant GBM

**Fig. 3:**
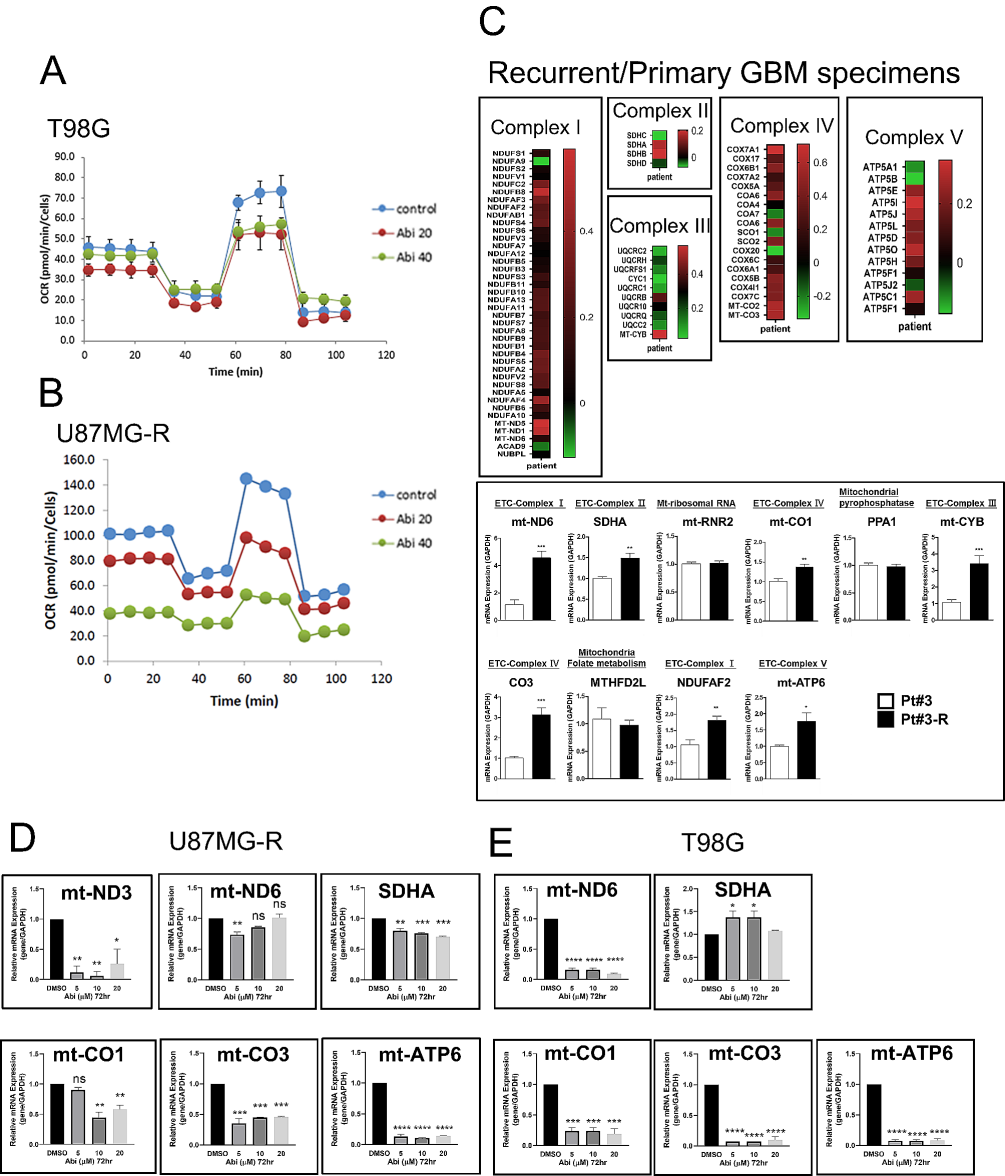
Effect of Abi on mitochondria respiration. After treatment with Abi for 72 h, T98G **A** and U87MG-R **B** cells were subjected to Seahorse XF Cell Mito Stress Test. Experiments, each containing two replicates, were performed independently three times. **C** Upper: Primary and recurrent specimens from GBM patients were homogenized and subjected to RNA-seq. Genes related to Complex I to V in mitochondria were shown in the heatmaps. Lower: RNA extracts from Pt#3 and Pt#3-R were prepared for RT-qPCR. **D** After treatment with Abi for 72 h, U87MG-R and T98G **E** cells were harvested for RNA extraction, followed by RT-qPCR. Experiments, each containing two replicates, were performed independently three times. (*P < 0.05, **P < 0.01, ***P < 0.001)

Recent discoveries have highlighted the role of mitochondrial activation in the development of drug resistance across various cancer types, including GBM. Our prior research indicated that mitochondria-mediated lipid metabolism is pivotal for the acquisition of TMZ resistance in GBM [24]. This study aims to investigate the effect of Abi on mitochondrial respiration within GBM cells. Through Seahorse analysis, we observed that Abi treatment significantly reduced mitochondrial respiration in T98G and U87MG-R cells **(**Fig. 3A**)**. Furthermore, an analysis of recurrent human GBM tissues revealed a notable upregulation of genes associated with mitochondrial respiration, including those encoding components of Complexes I to V, when compared to primary GBM samples **(**Fig. 3B**)**. Similarly, these mitochondrial genes, such as mitochondrially encoded NADH: ubiquinone oxidoreductase core subunit (mt-ND) 6, succinate dehydrogenase complex flavoprotein subunit A (SDHA), mitochondrially encoded cytochrome c oxidase I (mt-CO1), mitochondrially encoded cytochrome b (mt-CYB), mitochondrially encoded cytochrome c oxidase III (mt-CO3), ADH: ubiquinone oxidoreductase complex assembly factor (NDUFAF) 2, and mitochondrially encoded ATP synthase membrane subunit (mt-ATP) 6, exhibited a marked increase in expression in TMZ-resistant Pt#3-R cells relative to TMZ-sensitive Pt#3 cells **(**Fig. 3C**)**. In particular, Pt#3-R cells were maintained in the TMZ-containing medium for 12 months. Crucially, Abi treatment effectively downregulated several key mitochondrial genes, including mt-ND3, mt-ND6, mt-CO1, mt-CO3, and mt-ATP6, in both U87MG-R and T98G cells **(**Fig. 3D**)**. These findings suggest that Abi-mediated suppressing of GBM may involve the inhibition of mitochondrial respiration, offering new insights into the potential therapeutic action of Abi in overcoming drug resistance in GBM.

### Abi regulates mitochondria activity through cyclin-dependent kinase (CDK) 1

**Fig. 4:**
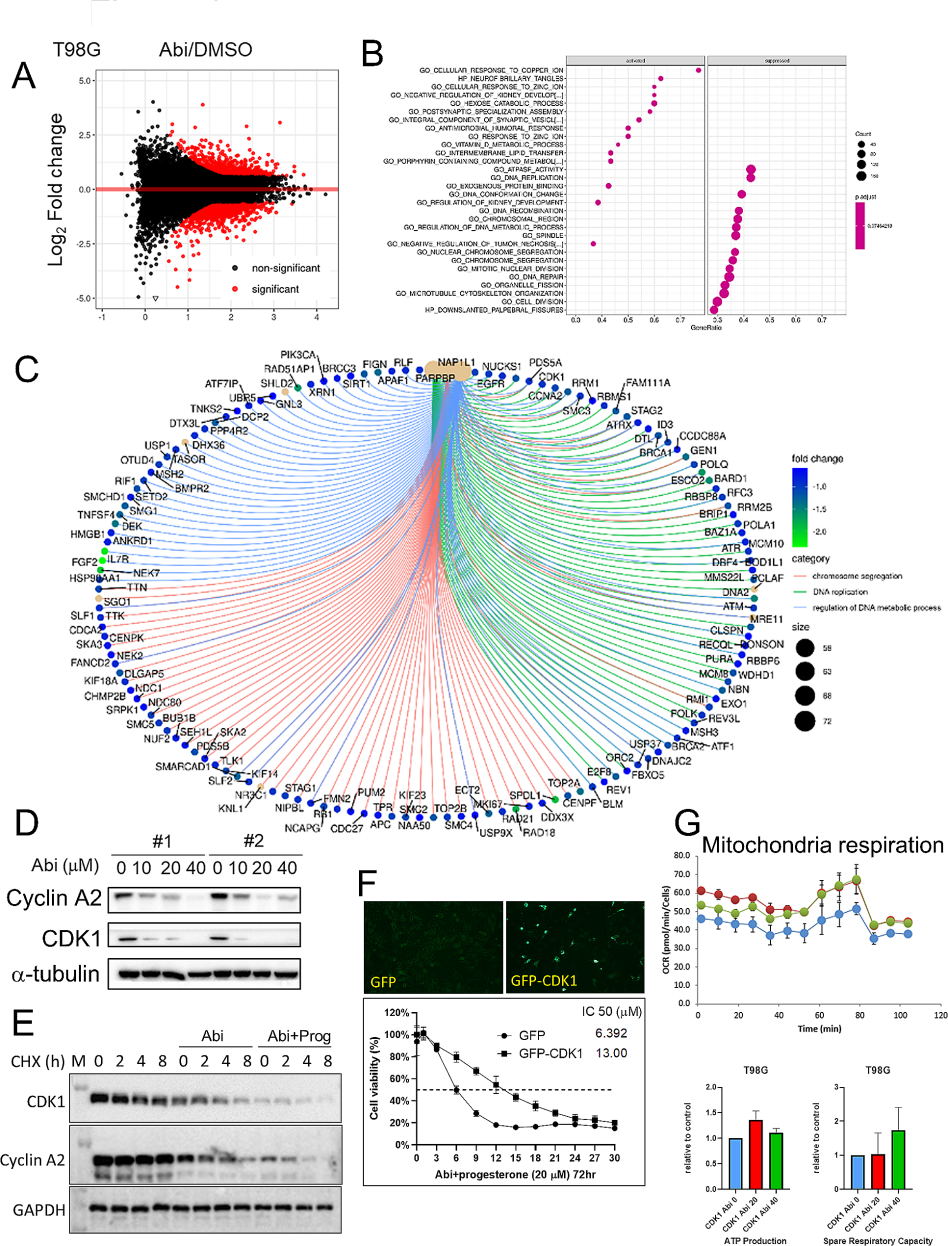
Abi suppresses mitochondria respiration through decreasing CDK1 expression. After treatment with Abi for 72 h, RNA extracts of T98G cells were subjected to RNA-seq. Genes influenced by Abi were shown **A** and functional clustered by Gene Set Enrichment Analysis (GSEA) **B**. **C** Abi-downregulated genes which were involved in G2/M transition and DNA replication were shown. **D** Western blotting for cyclin A2 and CDK1. **E** After treatment for 48 h, T98G cells were treated with 20 µM CHX for the indicated time interval, and cell lysates were collected for Western blotting. **F** Left: After transfection for 24 h, T98G cells were fixed and immunostained using the anti-GFP antibody. Right: After transfection for 24 h, cells were treated with Abi with or without Prog for 72 h. Cell viability was estimated using the CCK8 assay. Experiments, each containing three replicates, were performed independently three times. IC50 was calculated using the Prism software. **G** After transfection for 24 f and treatment with Abi for the additional 72 h, mitochondria respiration in T98G cells was estimated using Seahorse XF analyser. Experiments, each containing two replicates, were performed independently three times

To elucidate the mechanism behind Abi-mediated suppression of GBM, we conducted RNA-seq to map the gene expression landscape in T98G cells post-Abi treatment **(**Fig. 4A**)**. Following gene annotation, we identified both activated and suppressed cellular functions in Fig. 4B. Notably, the influence of Abi was predominantly seen in processes such as ATPase activation, DNA replication, chromosome segregation, and mitotic nuclear division, with a marked downregulation of genes involved in DNA replication and the G2/M phase transition **(**Fig. 4C**)**. Among these genes, we specifically validated the protein expression levels of cyclin A2 and CDK1 **(**Fig. 4D**)**. Intriguingly, CDK1, known for its role in regulating the G2/M phase transition, was also found to influence mitochondrial respiration [37]. This connection prompted further investigation into the involvement of CDK1 in the Abi-mediated inhibition of mitochondrial activation. In experiments where T98G cells were engineered to overexpress GFP-tagged CDK1, we observed an increased resistance to the combined treatment of Abi and Prog, suggesting a potential mechanism of action for therapeutic effect of Abi **(**Fig. 4E**)**. Moreover, CDK1 overexpression counteracted the Abi-induced suppression of mitochondrial respiration, further underscoring the importance of CDK1 in this context **(**Fig. 4F**)**. These findings collectively suggest that the capacity of Abi to suppress GBM growth and drug resistance might be attributed, in part, to its ability to inhibit CDK1-mediated mitochondrial respiration.

### Prog suppresses glycolysis through CDK1

**Fig. 5:**
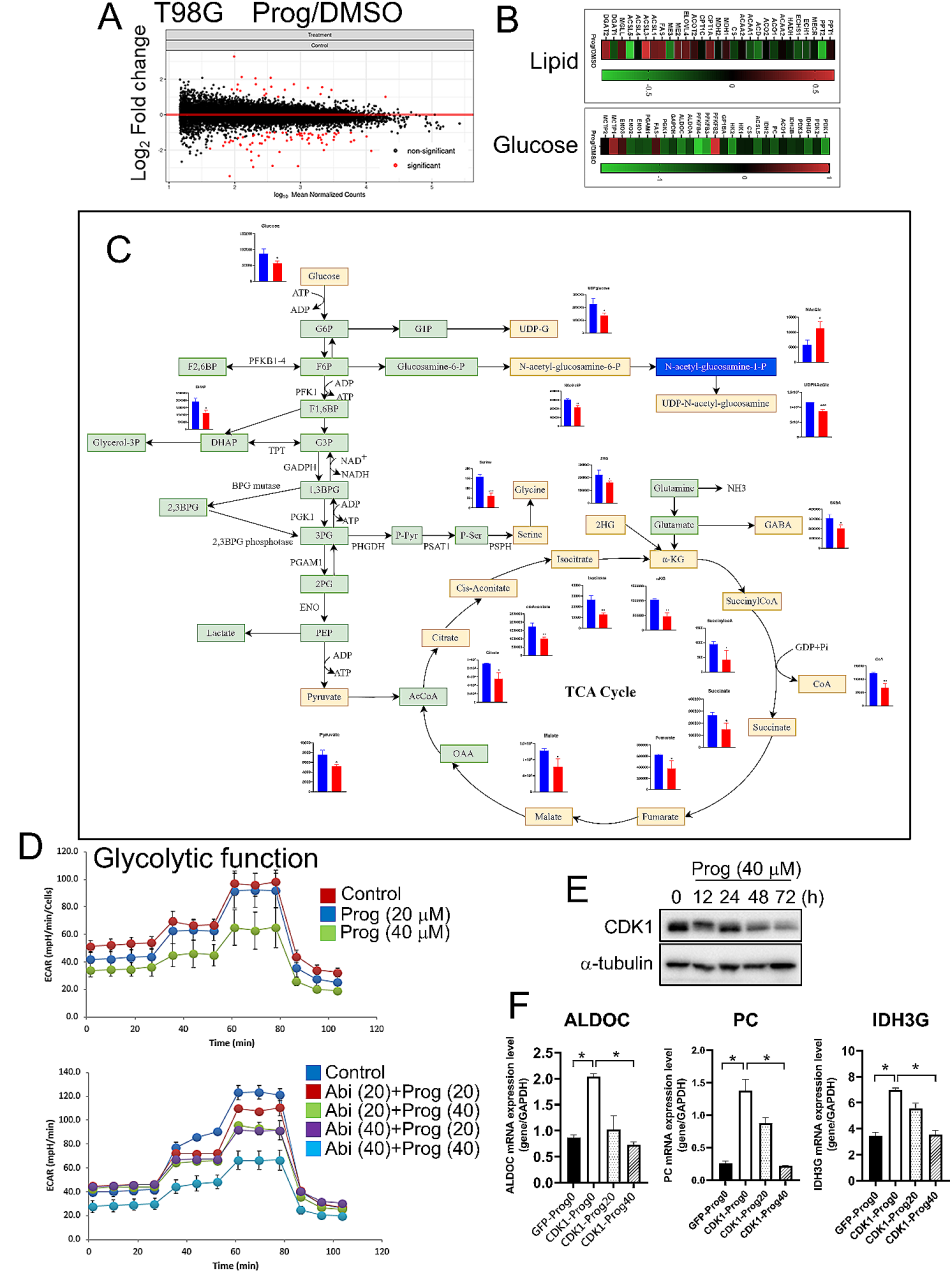
Effect of Prog on glycolysis in GBM cells. **A** After treatment with Prog (40 µM) for 72 h, RNA extracts of T98G cells were subjected to RNS-seq. Genes involved in lipid and glucose metabolism were shown in the heatmaps **B**. **C** After treatment with Prog for 72 h, protein lysates of T98G cells were subjected to metabolomics analysis using HPLC-MS. **D** After treatment for 72 h, glycolytic activity of T98G cells was estimated by the Seahorse Glycolysis Stress Test. Experiments, each containing two replicates, were performed independently three times. **E** Western blotting for CDK1. **F** After transfection for 24 h and treatment with Prog for the additional 72 h, RNA extracts were harvested for RT-qPCR targeting ALDOC, PC and IDH3G. Experiments, each containing three replicates, were performed independently three times. (*P < 0.05)

Herein, we aimed to elucidate the mechanism by which Prog enhances the suppression of GBM by Abi. Through RNA-seq, it was discovered that Prog predominantly downregulated genes associated with glucose metabolism rather than lipid metabolism **(**Fig. 5A**)**. This led us to conduct a targeted metabolomic analysis of metabolites involved in the progression from glucose metabolism to the tricarboxylic acid (TCA) cycle. Our findings indicated a significant reduction in the levels of key metabolites, including glucose, pyruvate, citrate, cis-aconitate, isocitrate, α-ketoglutarate (KG), succinyl-CoA, succinate, fumarate, and malate under Prog treatment **(**Fig. 5B**)**, highlighting the inhibitory effect of Prog on glucose metabolism as measured by the extracellular acidification rate (ECAR) **(**Upper panel, Fig. 5C**)**. Additionally, while Abi was observed to reduce glucose utilization, the presence of Prog further augmented this reduction in T98G cells **(**Lower panel, Fig. 5C**)**. Concurrent with the Abi-induced decrease in CDK1 expression, Prog also markedly lowered CDK1 protein levels **(**Fig. 5D**)**, underscoring the critical role of CDK1 in the therapeutic response. Further investigation revealed that the overexpression of CDK1 led to increased expression of key enzymes in glucose metabolism, including Aldoc aldolase C (ALDOC), pyruvate carboxylase (PC), and isocitrate dehydrogenase (IDH) 3G **(**Fig. 5E**)**. Notably, Prog significantly counteracted the CDK1-induced upregulation of these enzymes, suggesting a complex interplay where the combination of Abi and Prog disrupts GBM malignancy by targeting CDK1-mediated metabolic pathways. This comprehensive analysis reveals that the synergistic effect of Abi and Prog in combating GBM involves a multifaceted approach, including the inhibition of glucose metabolism and the modulation of CDK1 activity, providing a clearer understanding of their potential as a combined therapy in GBM treatment.

### Prog increases the infiltration of NK cells in the GBM

**Fig. 6:**
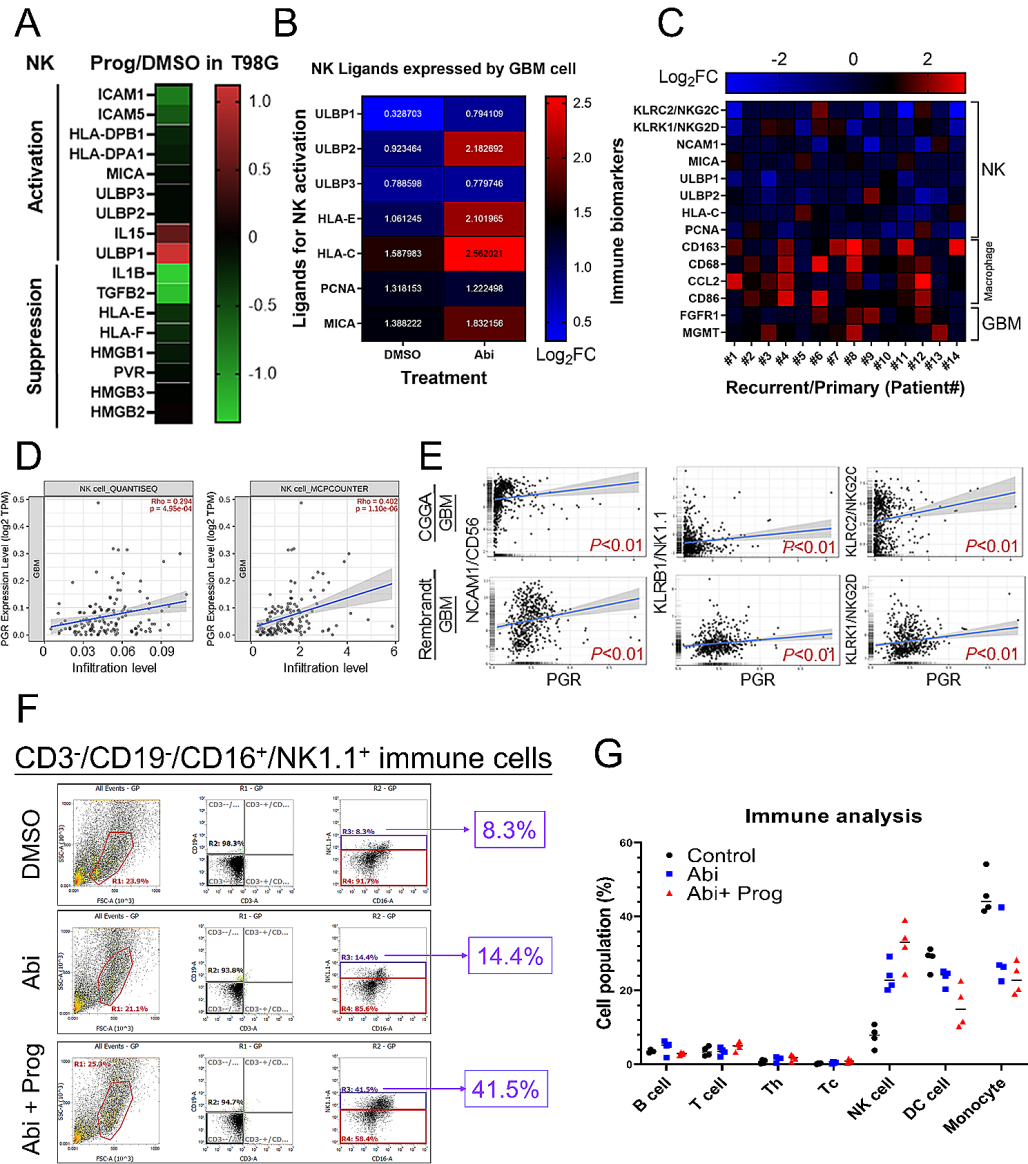
Effect of Prog on NK cell infiltration in GBM. **A** Genes related to NK cell activation and suppression were shown in the heatmap. **B** Abi-influenced NK ligands in the T98G cells. **C** Gene expression related to NK cell, macrophage and GBM in human GBM specimens. **D** NK cell infiltration was estimated using the TIMER website. **E** Correlation of gene expression was analysed using the Gliovis website. **F** After intracranial implantation with GL261-R cells for 7 days, experimental C57BL/6 mice were intraperitoneally injected with Abi or Abi + Prog twice per week for 3 weeks. Tumor in the brain was excised and subjected to immune-analysis using the indicated antibody by flow cytometry. Before gating for NK cells, cell suspension was gated for CD45^+^ cells. Four mice were included for each group. **G** Quantitative results for immune cells detected by flow cytometry. One symbol represents one mouse

Beyond attenuating glucose metabolism, RNA-seq analysis uncovered significant modulation of genes related to NK cell activity following Prog treatment in GBM cells. Specifically, RNA-seq revealed that genes implicated in the activation of NK cells, such as UL16 binding protein (ULBP) 1 and interleukin (IL) 15, were upregulated, while those associated with inhibiting NK cells, including IL1B and TGFB2, were downregulated **(**Fig. 6A**)**. Furthermore, Abi treatment was found to enhance the expression of genes essential for NK cell recognition, such as ULBP2, human leukocyte antigen (HLA)-C/E, and MHC class I polypeptide–related sequence (MIC) A **(**Fig. 6B**)**. Examination of tissue specimens from GBM patients revealed a notable decrease in NK-related markers in recurrent GBM compared to primary GBM specimens [24], contrasting with the upregulation of macrophage-related markers **(**Fig. 6C**)**. These findings indicate a correlation between the suppression of NK cell activity and GBM recurrence, suggesting that the combination of Abi and Prog could potentially counteract NK cell exhaustion. Notably, in GBM specimens analysed by the TIMER database, PgR expression positively correlated with the level of NK cell infiltration **(**Fig. 6D**)**. Furthermore, Gliovis analysis revealed that PgR expression exhibited a positive correlation with the expression of marker genes associated with active NK cells, including NCAM1/CD56, KLRB1/NK1.1, KLRC2/NKG2C, and KLRK1/NKG2D **(**Fig. 6E**)**. These results strongly suggest that PgR expression in GBM plays a pivotal role in recruiting NK cells, contributing to the establishment of a tumor-suppressive microenvironment. To investigate the impact of Abi and Prog on NK cell infiltration within GBM, we analysed the population of CD3^−^/CD19^−^/CD16^+^/NK1.1^+^ NK cells among CD45^+^ immune cells in implanted GL261-R GBM tissues from C57BL/6 mice **(**Fig. 6F-G**)**. The results indicated an increase in the NK cell population following Abi treatment, with Prog further enhancing its effect on NK cell accumulation, highlighting the potential of the combination of Abi with Prog in increasing NK cell accumulation, thereby augmenting GBM suppression.

### Prog facilitates NK cell therapy against GBM

**Fig. 7:**
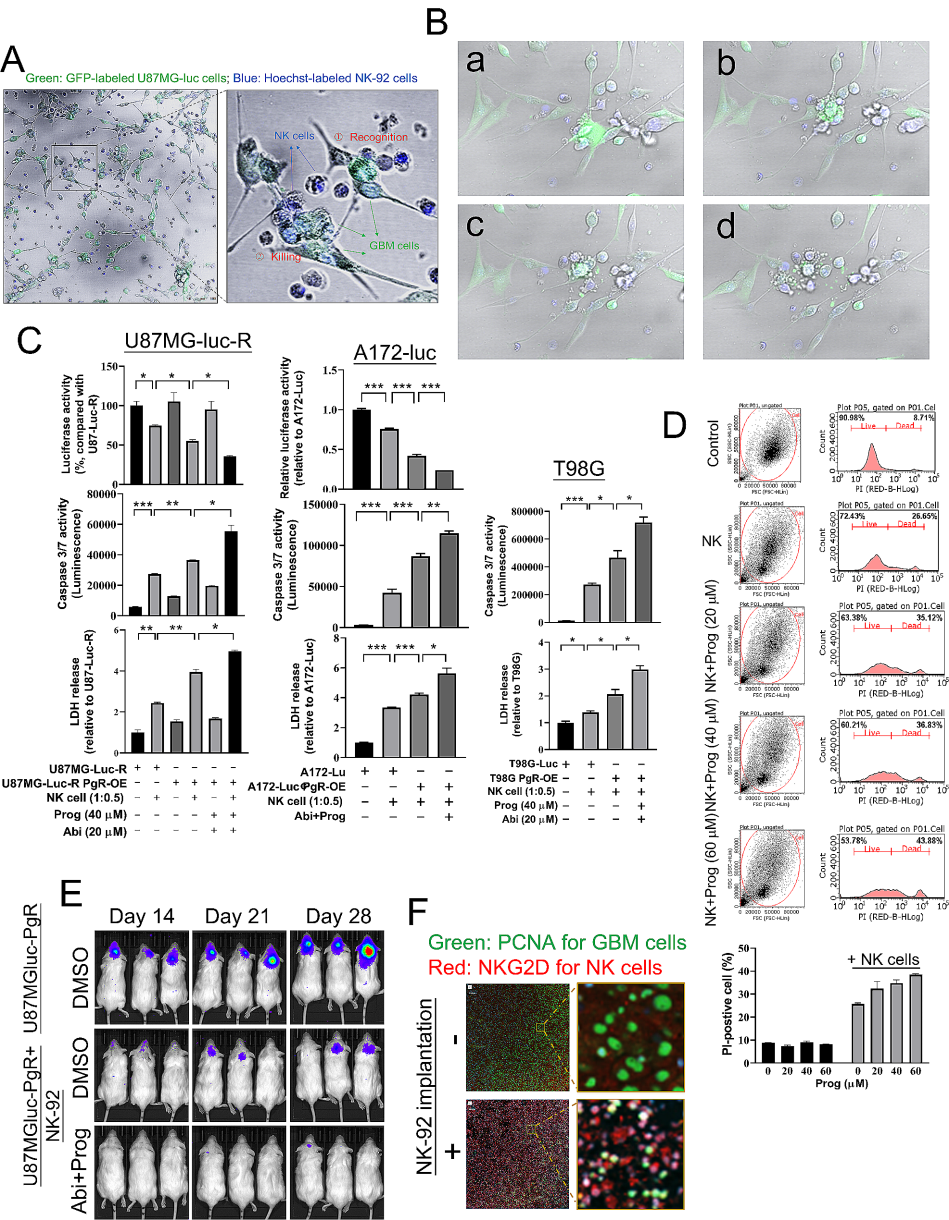
Effect of Prog on NK cell therapy against GBM. **A** GFP-expressed U87MGluc cells were co-cultured with Hoechst-stained NK-92 cells for 24 h. Cells were photographed by laser confocal microscope at the 24th h, and photographed every minute from 24th ~ 26th h **B**. **B** After co-culturing for 24 h, cells were monitored using a time-lapse microscope for 42 min. Four images were captured during the final 42 min of observation. a ~ d: from the beginning to the end. **C** GBM cells, U87MG-luc-R (left), A172 (middle) and T98G (right), were transfected with GFP-PgR and treated with the indicated drug for 48 h. Subsequently, GBM cells were co-incubated with NK-92 cells for 24 h. Cell viability of GBM was estimated using the luciferase assay (top). After co-culture, medium was collected for estimation of caspase 3/7 (middle) and LDH (bottom). Cell number: GBM: NK cells = 1:0.5. Experiments, each containing three replicates, were performed independently three times. (*P < 0.05, **P < 0.01, ***P < 0.001). **D** After co-culture for 24 h, NK-92 cell suspension was removed, and attached T98G cells were stained with PI, followed by the flow cytometric analysis. **E** After implantation with U87MG-luc or co-implantation with U87MG-luc and NK-92 cells, Abi and Prog were injected intraperitoneally on Day 6 (once per three days). IVIS images were acquired on the indicated day. Six mice were included for each group. **F** Brain slides for mice on Day 5 were prepared from the indicated mouse, and subjected to immunostaining. Green: PCNA; Red: NKG2D

To investigate whether Prog affects the anti-tumor properties of NK cells, we established a co-culture system using NK-92 and U87MG-luc cells. After 24 h of co-culturing, we observed the interaction between Hoechst-labelled NK-92 and GFP-labelled U87MG-luc cells. Based on morphological characteristics, we could distinguish NK-92-mediated recognition and killing targeting U87MG-luc cells **(**Fig. 7A**)**. The apoptosis of U87MG-luc cells induced by NK-92 was monitored using time-lapse microscopy **(**Fig. 7B, Supplementary Figure **S3** and Movie **S1)**. To evaluate the effect of Abi + Prog on NK cell-mediated tumor killing efficacy, we first assessed the impact of PgR overexpression, which is required by Prog to initiate the genomic pathway, in a co-culture system of NK-92 and U87MG-luc cells. In Supplementary Figure **S4A**, we found that both PgR overexpression and Abi + Prog treatment enhanced NK-92 cell-mediated killing of U87MG-luc cells, as shown by the luciferase assay, with similar efficacy. Notably, PgR overexpression further enhanced the efficacy of Abi + Prog treatment on NK cell therapy in vitro. Additionally, NK-92 cells significantly reduced the viability of PgR-expressing U87MG-luc cells, and compared to Abi treatment alone, the combination with Prog further enhanced NK-92-mediated tumor suppression (Supplementary Figure **S4B**). We validated this phenomenon in U87MG-luc-R, A172-luc, and T98G cells **(**Fig. 7C**)**. In U87MG-luc-R and A172-luc cells, PgR overexpression improved NK-92-mediated tumor killing, and Abi + Prog treatment further enhanced this effect. Levels of lactate dehydrogenase (LDH) and caspase 3/7, markers of apoptosis, increased consistently with reduced cell viability. Although T98G cells lack luciferase expression, Abi + Prog treatment still enhanced NK cell therapy as evidenced by significant upregulation of LDH and caspase 3/7 levels **(**Fig. 7C**)**. In particular, we excluded that the upregulation of LDH and caspase 3/7 was not caused by the expansion of NK-92 cells after co-culture for 24 h (Supplementary Figure **S4C**). In Fig. 7D, NK-92 cells induced 26.65% apoptosis in T98G cells determined by PI staining and flow cytometry, with Prog treatment further increasing apoptosis dose-dependently to 43.88%. In the orthotopic mouse model, PgR overexpression did not affect U87MG-luc-R cell growth in vivo but enhanced NK-92 cell efficacy, which was also achieved with Abi + Prog treatment. Particularly, when U87MG-luc-R cells overexpressed PgR, Abi + Prog treatment maximally enhanced NK cell therapy (Supplementary Figure **S4D**). Notably, co-implantation with U87MG-luc and NK-92 cells robustly suppressed the growth of U87MGluc-induced GBM in vivo, with the efficacy further enhanced by the combination of Abi and Prog **(**Fig. 7E**)**. Particularly, NK-92 cells implanted alongside U87MG-luc cells were detected in the GBM on Day 5 using immunostaining targeting the NK cell receptor, NKG2D **(**Fig. 7F**)**. These results indicate that NK cells can inhibit GBM development, and Abi combined with Prog enhances the GBM-suppressive efficacy of NK cells.

## Discussion

The investigation into the synergistic effects of Prog and Abi on GBM uncovers promising strategies for overcoming drug resistance, particularly in TMZ-resistant strains. By combining capability of Abi to induce ER stress with Prog-mediated metabolic and apoptotic influences [6], we observed a marked enhancement in therapeutic efficacy. This synergy is further highlighted by the significant reduction in MGMT expression and mitochondrial respiration in GBM cells, showcasing a potent approach to inhibit tumor survival mechanisms. Additionally, our study reveals an intriguing aspect of Prog in modulating the immune response, particularly through the upregulation of NK cell activity, which could contribute to overcoming GBM malignancy.

The exploration into the effects of medroxyprogesterone acetate (MPA) across various studies offers valuable parallels to our own investigation into the combination of Prog and Abi for TMZ-resistant GBM [22]. The former studies shed light on the capacity of hormonal treatments to inhibit glioma cell proliferation and invasion, suggesting their significant role in cancer therapy. Specifically, the anti-tumor, anti-inflammatory, and neuroprotective properties of Prog, as demonstrated in these studies, support the hypothesis that hormonal interventions can substantially benefit GBM treatment outcomes [9, 11, 38, 39]. This aligns with our findings that Prog, in combination with Abi, significantly enhances therapeutic efficacy against TMZ-resistant GBM cells, underscoring the potential of hormonal strategies in overcoming treatment resistance. In comparing the effectiveness of Prog versus dexamethasone (Dexa) in extending survival and preserving neurological function in rats with orthotopic GBM allografts [38], Prog given its anti-inflammatory, antiedema, and neuroprotective properties, might be a viable alternative to Dexa for managing peritumoral brain edema [40]. The findings indicate that Prog significantly extended overall survival, better preserved neurological function, and maintained body weight compared to Dexa, supporting the potential use of Prog as a corticosteroid-sparing agent in brain tumor management.

Another interesting study also indicated the anti-tumor efficacy of Prog. The tumor-promoting effects of the beta-subunit of human chorionic gonadotropin (beta-hCG) was limited by Prog via non-nuclear receptors [41], highlighting how Prog mediates its anti-tumor effects independently of nuclear receptors. Prog induced apoptosis in beta-hCG responsive tumor cells and countered the pro-tumorigenic signals of beta-hCG, especially in the absence of ovaries, mimicking the post-menopausal state. RNA-seq profiling revealed molecular signatures that underline the anti-tumor effects of prog, suggesting its potential therapeutic application in cancers expressing beta-hCG, particularly in post-menopausal women.

Prog-mediated suppression of mitochondrial functions and glycolytic metabolism in GBM suggests its influence on energy metabolism within GBM cells. Another study similarly demonstrated that Prog disrupted cancer cell metabolism by inhibiting mitochondrial activity and reducing glycolysis [39], aligning closely with our research on the synergistic effects of Prog and Abi. These findings collectively bolster the rationale for integrating Prog into GBM treatment, highlighting the significance of metabolic interventions in combating this challenging disease.

RNA-seq analysis revealed significant changes in genes related to NK cell activity following Prog treatment in GBM cells, with upregulation of NK cell activation genes and downregulation of inhibitory genes. Abi treatment further enhanced NK cell recognition genes. This aligns with literature discussing the influence of Prog on NK cells in pregnancy contexts, highlighting mechanisms such as IL15 production and interactions with stromal cells [42]. While we extend this understanding to GBM treatment, emphasizing a potential correlation between NK cell activity suppression and GBM recurrence, it parallels the broader role of Prog in modulating NK cell function through cytokine signaling and indirect mechanisms. The findings suggest a novel therapeutic approach to boost NK cell activation and accumulation for GBM suppression, bridging insights from reproductive biology to cancer immunotherapy.

In conclusion, the combination of Abi and Prog shows promising synergistic effects in targeting TMZ-resistant GBM. Prog enhances the inhibitory efficacy of Abi on GBM growth by downregulating the DNA repair protein MGMT. Abi-mediated suppression of mitochondrial respiration and CDK1-mediated pathways, coupled with Prog-mediated inhibition of glucose metabolism via CDK1 modulation, presents a multifaceted approach to combat GBM malignancy. Moreover, this combined therapy modulates the immune microenvironment by enhancing NK cell infiltration and activation while reducing dendritic cells and monocytes, potentially overcoming NK cell exhaustion associated with GBM recurrence. Future research should focus on clinical validation, exploring additional mechanisms, and developing personalized treatment strategies to maximize therapeutic outcomes in GBM patients.

### Electronic supplementary material

Below is the link to the electronic supplementary material.

Supplementary Material 1

Supplementary Material 2

## Data Availability

Data available upon request to the author.
