# Supplementary material for: Progesterone boosts abiraterone-driven target and NK cell therapies against glioblastoma
Source: J Exp Clin Cancer Res. 2024 Aug 6;43:218. doi: 10.1186/s13046-024-03144-2 (PMC11302026; doi:10.1186/s13046-024-03144-2)
Supplement: Supplementary file 1 — Supplementary Material 1 [file 13046_2024_3144_MOESM1_ESM.docx]

**Supplementary information**

**Progesterone Boosts Abiraterone-Driven Target and NK Cell Therapies Against Glioblastoma**

Hsien-Chung Chen^1,2,3#^, Hong-Yi Lin^2,4#^, Yung-Hsiao Chiang^2,4,5,6^, Wen-Bin Yang^1,7^, Chung-Han Wang^1^, Pei-Yu Yang^1^, Siou-Lian Hu^1^, Tsung-I Hsu^1,4,7,8,9,10^*

^1^Ph.D. Program in Medical Neuroscience, College of Medical Science and Technology, Taipei Medical University and National Health Research Institutes, Taipei, Taiwan.

^2^Taipei Neuroscience Institute, Taipei Medical University, Taipei, Taiwan

^3^Department of Neurosurgery, Shuang Ho Hospital, Taipei Medical University, Taipei, Taiwan

^4^Research Center for Neuroscience, Taipei Medical University, Taipei, Taiwan

^5^Department of Neurosurgery, Taipei Medical University Hospital, Taipei Medical University, Taipei, Taiwan

^6^Department of Surgery, College of Medicine, Taipei Medical University, Taipei, Taiwan

^7^International Master Program in Medical Neuroscience, College of Medical Science and Technology, Taipei Medical University, Taipei, Taiwan

^8^TMU Research Center for Drug Discovery, Taipei Medical University, Taipei, Taiwan.

^9^Ph.D. Program in Drug Discovery and Development Industry, College of Pharmacy, Taipei Medical University

^10^TMU Research Center of Cancer Translational Medicine, Taipei, Taiwan

Hsien-Chung Chen^#^ and Hong-Yi Lin^#^ contribute to this work equally.

*Corresponding author:

Tsung-I Hsu*: Ph.D. Program in Medical Neuroscience, College of Medical Science and Technology, Taipei Medical University and National Health Research Institutes, Taipei, Taiwan; E-mail: [dabiemhsu@tmu.edu.tw](mailto:dabiemhsu@tmu.edu.tw)

**Supplementary Figure S1. Effects of Abi, Prog and the combination of Abi with Prog on the proliferation of U87MG cells.** After treatment for 72 hours, cells were harvested for CCK8 assay.


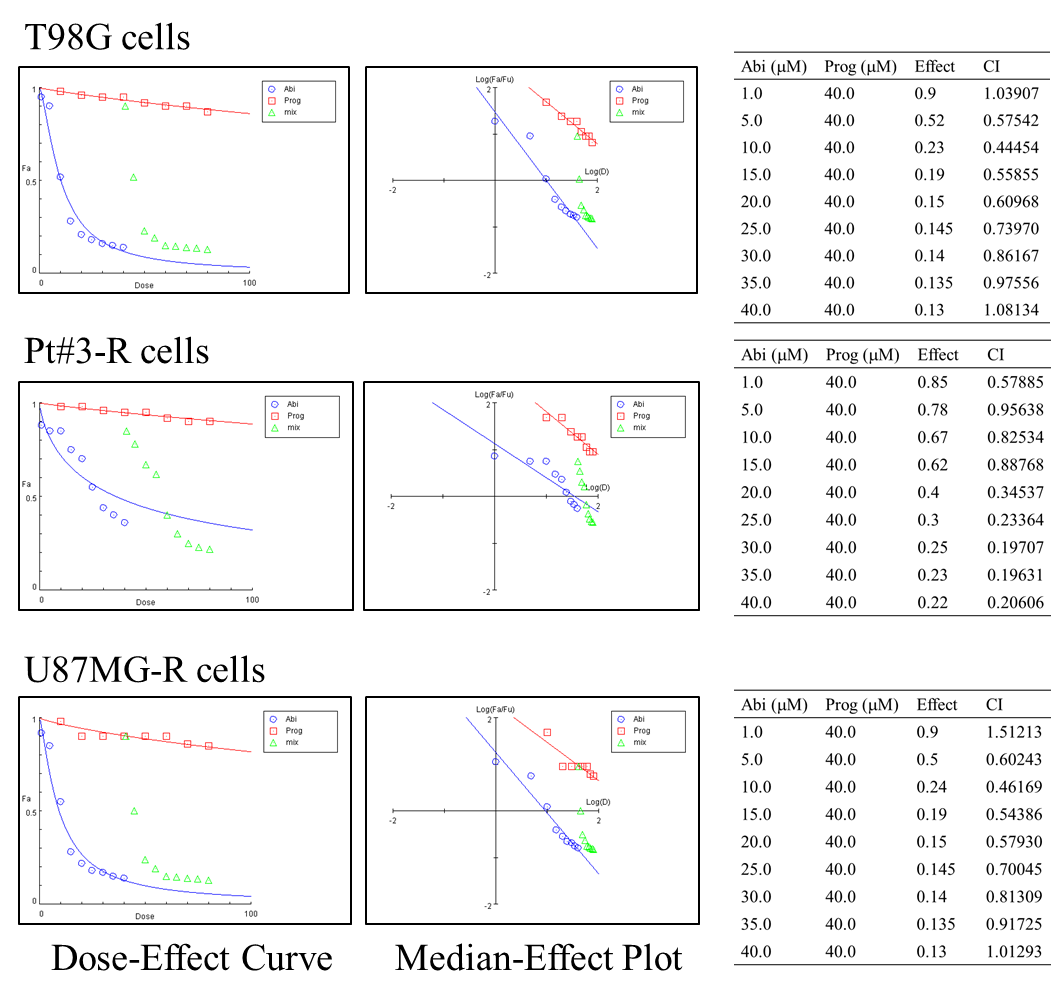


**Supplementary Figure S2. The synergy for GBM treatment was analysed using the CompuSyn software.** CI was shown in the right panel.


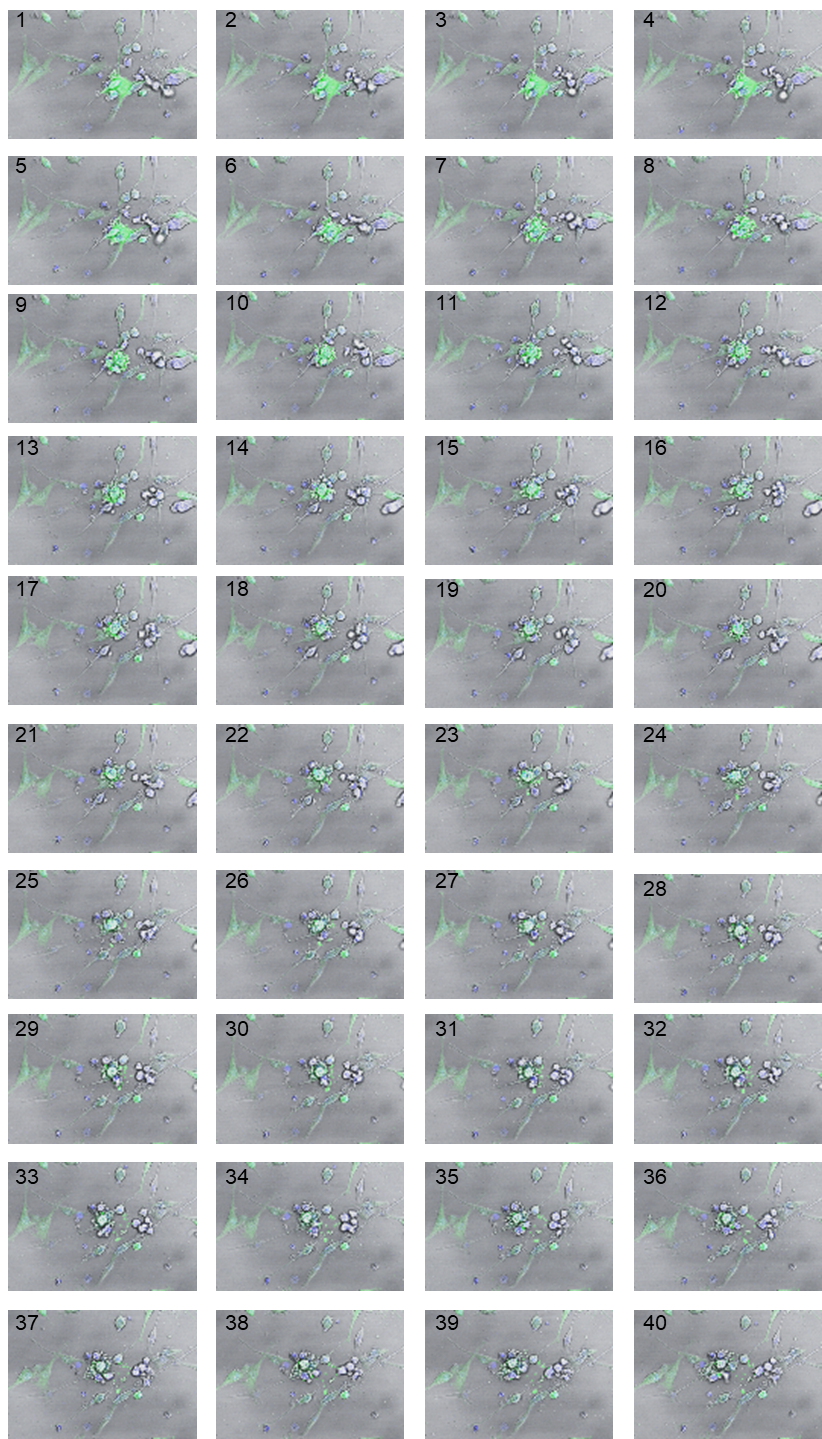


**Supplementary Figure S3. Co-culture of GFP-expressed U87MG-luc cells with Hoechst-stained NK-92 cells.** After co-culture for 24 hours, cell-cell interaction was monitored under the time-lapse microscope. Forty-two images of the latest 42 min (1 image per min).


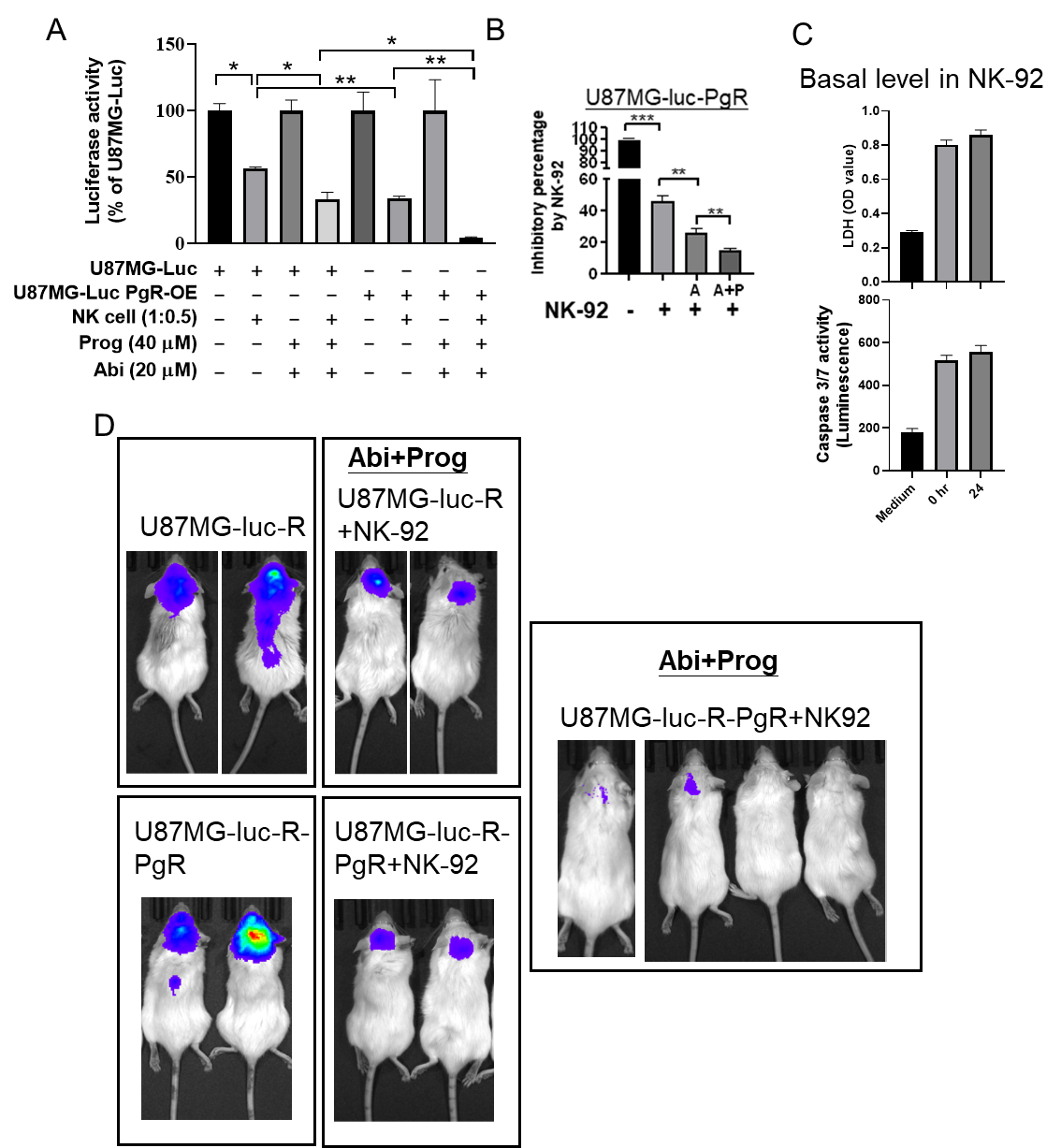


**Supplementary Figure S4. Effect of PgR overexpression and Abi+Prog treatment on NK-92 cells-mediated tumor killing of GBM. A-B.** U87MG-luc cells were transfected with GFP-PgR and treated with the indicated drug for 48 h. Subsequently, U87MG-luc cells were co-incubated with NK-92 cells for 24 h. Cell viability of U87MG-luc was estimated using the luciferase assay. (**P*<0.05, ***P*<0.01, ****P*<0.001). C. Basal levels of LDH and caspase 3/7 in NK-92 cells. D. After implantation with U87MG-luc-R/U87MG-luc-R-PgR or co-implantation with U87MG-luc/U87MG-luc-R-PgR and NK-92 cells, Abi and Prog were injected intraperitoneally on Day 6 (once per three days). Four mice were included for each group. IVIS images were acquired on Day 28.
