# Supplementary figures and images for: Progesterone boosts abiraterone-driven target and NK cell therapies against glioblastoma
Source: J Exp Clin Cancer Res. 2024 Aug 6;43:218. doi: 10.1186/s13046-024-03144-2 (PMC11302026; doi:10.1186/s13046-024-03144-2)

## Slide 1
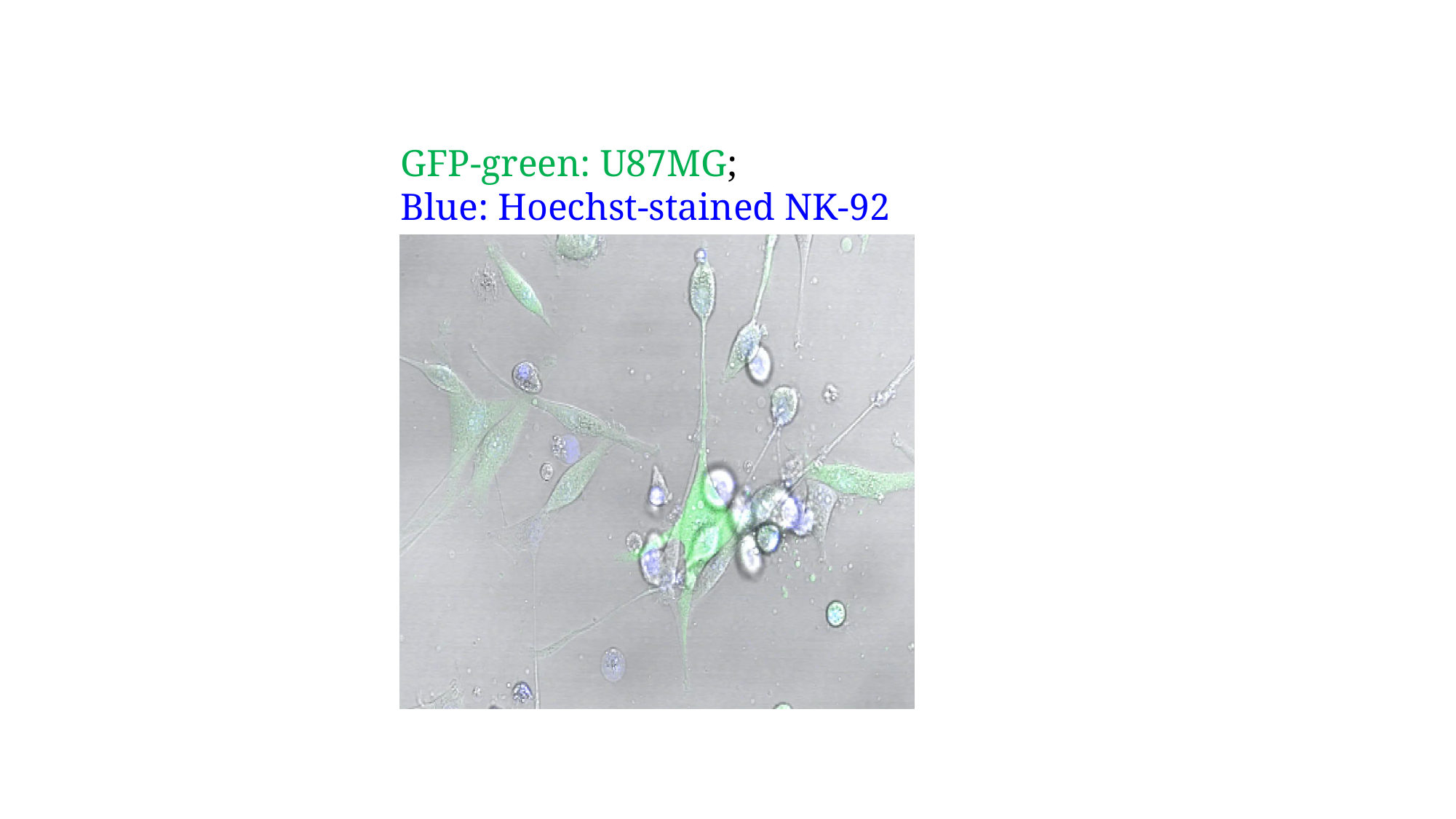

GFP-green: U87MG;
Blue: Hoechst-stained NK-92

Supplement: Supplementary file 2 — Supplementary Material 2 [file 13046_2024_3144_MOESM2_ESM.pptx]
